# Rush Medical College

**Published:** 1845-12

**Authors:** 


RUSH MEDICAL COLLEGE.

The present Session of this Institution has opened under more favorable prospects than any of the preceding, both as regards the advantages offered for teaching, and the number and character of the class in attendance. During the past summer, arrangements were made for forming the various cabinets in connexion with the various departments. That of Materia Medica is already complete, embracing nearly every substance used in Medicine in its pure state, and is, in every respect, one of the most perfect possessed by any Medical School in the country. That of tumours and morbid specimens has also been greatly enlarged, and now embraces specimens of the pathological anatomy of a great number of diseases. The cabinet of mineralogy, and that of anatomical preparations, are now being arranged, and a sufficient number is already posessed for each, to render them highly valuable and instructing. These, in connexion with the advantages heretofore offered, place the School on an equality with many long established. The present class shows an increase over that of last session, although the rule has been adopted that the lecture fees must be paid in cash, or secured by good indorsed notes, payable within a year.

The effects of this regulation have already been most evident and beneficial. The medical public are perhaps not sufficiently aware of all the arts used by many medical schools for the purpose of securing the attendance of students, and swelling the numbers of the class. Travelling agents are employed to seek them out, promise them easy terms, and draw into the study those engaged in other occupations. These soon go out as practitioners, either with or without diplomas, and the evil inflicted upon the profession by such means, exceeds that of every variety of quackery. Against such practices, we determined from the beginning to set our example, relying upon the good judgment of the medical public to sustain us.

Great effort will be made to secure for the next session every advantage enjoyed by the oldest medical schools. Arrangements have been already made by which a library, extensive and well selected, will be attached to the institution, and others are in progress for securing hospital instruction. Numerous cases and operations have been exhibited before the class in the surgical clinic, but they resemble for the most part so nearly those heretofore reported in this journal, that it is not thought necessary to report the clinical lectures upon them. D. B.

